# Environment and Diet Influence the Bacterial Microbiome of *Ambigolimax valentianus*, an Invasive Slug in California

**DOI:** 10.3390/insects12070575

**Published:** 2021-06-23

**Authors:** Denise Jackson, Mia R. Maltz, Hannah L. Freund, James Borneman, Emma Aronson

**Affiliations:** 1 Department of Microbiology and Plant Pathology, University of California, Riverside, CA 92521, USA; djack006@ucr.edu (D.J.); hfreu002@ucr.edu (H.L.F.); borneman@ucr.edu (J.B.); 2 Natural Science Division, Porterville College, Porterville, CA 93257, USA; 3 Center for Conservation Biology, University of California, Riverside, CA 92521, USA; maltz@ucr.edu; 4 Division of Biomedical Sciences, University of California, Riverside, CA 92521, USA; 5 Genetics, Genomics, and Bioinformatics Program, University of California, Riverside, CA 92521, USA

**Keywords:** terrestrial slug, gastropod, bacteria, microbiome, host

## Abstract

**Simple Summary:**

Slugs are significant pests, physically damaging plants from their voracious appetite as well as dispersing bacteria which could be harmful to plants and humans. They produce substantial economic costs, both as a direct result of plant destruction, and indirectly through attempts of pest control. This study explored the ecological aspects of the bacterial microbiome of *Ambigolimax valentianus*, a slug invasive to California. We identified a core microbiome in *A. valentianus* and found that their bacterial microbiome can be influenced and may depend substantially on both diet and environment. We also found that *A. valentianus* slugs harbor ecologically important bacteria, therefore their dispersal could have environmental and agricultural implications for both crop health and plant science. Future studies that provide a better understanding of the slug bacterial microbiome may be an important step in the development of comprehensive slug management.

**Abstract:**

*Ambigolimax valentianus* is an invasive European terrestrial gastropod distributed throughout California. It is a serious pest of gardens, plant nurseries, and greenhouses. We evaluated the bacterial microbiome of whole slugs to capture a more detailed picture of bacterial diversity and composition in this host. We concentrated on the influences of diet and environment on the *Ambigolimax valentianus* core bacterial microbiome as a starting point for obtaining valuable information to aid in future slug microbiome studies. *Ambigolimax valentianus* were collected from two environments (gardens or reared from eggs in a laboratory). DNA from whole slugs were extracted and next-generation 16S rRNA gene sequencing was performed. Slug microbiomes differed between environmental sources (garden- vs. lab-reared) and were influenced by a sterile diet. Lab-reared slugs fed an unsterile diet harbored greater bacterial species than garden-reared slugs. A small core microbiome was present that was shared across all slug treatments. This is consistent with our hypothesis that a core microbiome is present and will not change due to these treatments. Findings from this study will help elucidate the impacts of slug-assisted bacterial dispersal on soils and plants, while providing valuable information about the slug microbiome for potential integrated pest research applications.

## 1. Introduction

With the advances of next-generation sequencing, research focused on host–microbiome systems has expanded and now includes a wider range of plants and animals. All invertebrates associate with bacterial communities, which form a component of their microbiome. Notably, invertebrates’ microbiomes are often overlooked. Bacteria associated with invertebrates play many roles in association with their hosts—including protection or supporting overall health and fitness—or have negative implications for the host, and may, therefore, be ecologically important [1,2]. Invertebrate systems have been shown to be excellent models for the study of host–bacterial associations, partially due to their smaller size and rather uncomplicated gut communities [3]; yet, to date, little work has been conducted in malacological (the study of mollusks) research focused on bacteria associated with whole terrestrial slugs [4].

Several observations concerning the life-history traits (reproduction, growth, distribution, seasonal variation, and fitness) of slugs have been explored [5,6,7,8,9]. Disruption of these traits may lead to decreased slug fitness or slug death; therefore, any substances that affect these traits should be explored as possible alternatives for slug control. Few studies have focused on the role of the gastropod immune response [10,11] and how bacteria may affect these traits. The majority of terrestrial gastropod microbiome research focuses on the guts or feces of this invertebrate [12,13,14,15,16,17,18,19,20,21,22,23,24,25,26]; however, the cellular and humoral components that make up the slug innate immune response are found in the slug’s open circulatory system (hemolymph) throughout the slug’s body [27,28,29]. The characterization of the bacterial community of whole slugs may provide knowledge about the slug immune response and possibly help identify the bacterial players involved. Additionally, bacteria have been shown to be affective against other pest species such as bollworms, cotton leaf worms, nematodes, mussels, and snails [30,31,32,33,34,35,36]. Information regarding the bacterial players involved in slugs would be useful for future slug life-history studies and may aid in the development of bacterial biocontrol against slugs.

Slugs may serve as vectors for transporting microorganisms from place to place. Therefore, many slugs that are considered invasive could harbor or translocate a variety of exotic or pathogenic microorganisms within their microbiome. Horticulturalists, agronomists, and land managers recognize the need to effectively control slug populations in an ecologically sound manner. Bacterial communities within organisms have notable functions to their hosts survival, and in some cases, provide its host with insecticide resistance [37,38,39,40]. Furthermore, these microbes can offer protection to its host from its natural enemies [41,42,43]. The interactions between slugs, bacteria, and their environment can vary; characterizing the bacterial community within—and among—slugs is an important step for elucidating the nature of these various interactions [44]. Given their fundamental role, slugs provide an exemplary system for addressing questions concerning composition, function, and diversity of this malacological microbiome [3,45].

Slugs have the capacity to thrive in a range of conditions. Indeed, their success is attributed to mucus production that deters predators, as well as high reproduction rates and adaptable appetites [4]. They are major pests of plant nurseries and several agricultural crops, including corn, soybean, wheat, brassicas, leafy vegetables, and strawberry crop systems [46]. Slugs target a variety of plants and grasses, often by killing seedlings outright, causing considerable amounts of economic damage in California arable and horticultural crops, commercial nurseries, and home gardens [4,47].

Most slug species found in California nurseries are invasive, with many having been transported long distances. Known invasive slug species in California include members of genera *Deroceras* and *Arion*, as well as the species *Milax gagates* and *Ambigolimax valentianus*, all originating from Europe [4,46]. Although individual slugs do not move rapidly per se, trade in horticultural commodities has facilitated their spread. As a result, additional exotic slug species, or species not considered to be endemic to California, are likely to appear there in the near future [4,46].

Malacological studies have yet to determine the bacterial microbiome of whole slugs, and more importantly, address the context wherein diversity of the slug bacterial microbiomes is being shaped. The bacterial microbiome of terrestrial gastropods has displayed many important functions, including playing a vital role in digestion and having metabolic capabilities, enzymatic activity, and biochemical activity reflective of plant biomass degradation and the breakdown of lignocellulose [12,13,14,15,16,17,18,19,20,21,22,23,24,25,26]. Their bacterial microbiome also responds to changes in their diet and environment, which has shown to affect their weight and health [14,21,48,49,50]. To gain an understanding into the slug’s bacterial ecological relationships, we investigated the bacterial microbiome of *Ambigolimax valentianus*, a slug invasive to California, using sterile microcosm experiments. Moreover, to address some factors shaping slug bacterial microbiomes, we evaluated whether the bacterial microbiome of *A. valentianus* can be influenced by changing their diet and environment. To elucidate the impacts of slug-assisted bacterial dispersal on soils and plants, we hypothesized that (1) The overall bacterial communities of *A. valentianus* can be manipulated via their environment and diet, while (2) a core bacterial community creates the basis for future studies of slug microbiomes on host physiology, but will also provide useful information for Integrated Pest Management applications.

## 2. Materials and Methods

### 2.1. Study Species

*Ambigolimax valentianus* evolved in Europe and is invasive in California, geographically distributed throughout at least 29 counties in California [46]. One distinguishing morphological characteristic of *A. valentianus* is the presence of two distinct lines extending down their back along the entire length of their body. These slugs are wide-scale pests, feeding on plants and decomposing wood [46].

### 2.2. Sample Collection

Adult slugs were collected with sterile gloves from shaded areas beneath flats and pots of various plants from Louie’s Nursery, a market garden located in Riverside, California in April 2017. Upon collection, slugs were placed in sterile 15 mL conical tubes and subsequently returned to the laboratory at the University of California, Riverside, for downstream analyses. For characterizing the initial slug bacterial microbiome, five of these slugs were frozen immediately. The remaining 15 were divided into three sterile microcosms, consisting of five slugs each.

In addition to slugs collected from the garden, slugs were also reared in the laboratory. The eggs from previously collected *A. valentianus* were gathered and kept in a dish with moist paper towels at room temperature (~20–23 °C) due to their vulnerability to increased heat and cold [51,52]. The juvenile slugs that emerged from the eggs were transferred to new dishes, kept moist, and fed with carrots and wet dog food. Slugs are usually characterized by a juvenile or adult stage (sexually maturity), the latter of which can take up to a few months to reach [47,53,54]. Laboratory-reared slugs were given 4–6 months of growth to reach maturity to be consistent with the size of the adult slugs collected from the garden. Ten randomly selected laboratory-reared slugs were divided; five were frozen for initial bacterial microbiome analyses and the remaining five were placed into a sterile microcosm.

### 2.3. Experimental Design

Each microcosm was composed of sterile, autoclaved paper towel, initially moistened with ~5 mL of sterile water; thereafter, small amounts of sterile water were added to each microcosm to maintain consistent moisture. All slugs were fed ad libitum with a sterile diet composed of a mixture of autoclaved carrots, bran, and nutrient agar. To maintain sterility, each microcosm was only opened in a biosafety cabinet; sterile forceps were used for feeding, as well as slug placement or removal.

Laboratory-reared slugs were initially fed non-sterile dry and wet canned dog food and carrots. Five of those slugs were placed into a sterile microcosm, as described above. Some species of parasitic nematodes are natural enemies of slugs. They enter the slug through natural openings and release pathogenic bacteria into the slug mantle. Symptoms of nematode infection, such as swelling of the mantle and death, can appear as early as 4 days [55,56,57,58,59,60]. To prevent using slugs naturally infected with nematodes, slugs were housed in sterile microcosms for two weeks before DNA analyses.

### 2.4. Sample Processing

Whole slug tissues were prepared for DNA extraction in 15 mL conical tubes by blending each slug with sterile water using 14 G, 16 G, and 18 G needles (in sequential order) to create a slug mixture. The amount of sterile water added was determined by slug weight. DNA extraction of the slug mixtures was performed using the MoBio PowerSoil^®^ DNA extraction kit (Qiagen Inc., Valencia, CA USA). An aliquot of 250 µL of each slug mixture was used in lieu of the 0.25 g of soil called for in the kit protocol. Slug DNA extracts were amplified by PCR to capture the full variety of the 16S rRNA genes within each sample. These PCR extracts were sequenced using the Illumina MiSeq (Illumina, Inc., San Diego, CA USA) system (2 × 300) allowing for the sequencing of a ~450 bp section of the 16S V3 and V4 region of the 16S rRNA gene. The sequences were multiplexed using barcoded indexes and primers from the Illumina Nextera XT kit [61].

### 2.5. Data Analysis and Bioinformatics

To examine the core bacterial microbiome, as well as the relationship between the core and communities from environment and diet, sequences were processed with Quantitative Insights Into Microbial Ecology [62]. This approach was used to determine the relationship between bacterial microbiome communities and host diet, rearing, and sterility variables. We removed low-quality and chimeric sequences and computed core microbiomes in QIIME (Open-source software, Caporaso Lab- Flagstaff, AZ USA and Knight Lab- San Diego, CA USA); we amplified sterile PCR-grade water, as a negative control, which was processed alongside the slug DNA samples. After samples were extracted, amplified, and sequenced, any OTUs that were present in the negative controls were removed from downstream analyses. We define the core bacterial microbiome of slugs as the bacteria commonly detected among all sampled slugs [63].

Bacterial alpha diversity of the samples was determined using rarefied OTU counts via the ‘rrarefy’ function in the vegan package for R [64]. Species richness was calculated using raw OTU counts via the ‘specnumber’ function in the vegan package for R [64]. Alpha diversity and species richness across treatment conditions and treatment sources were compared, respectively, using Wilcoxon tests, performed with the ‘compare_means’ function in the ‘ggpubr’ package for R [65].

To determine if bacterial beta diversity was different between samples, unweighted Unifrac distance matrices were created and used to compare community samples. To visualize and explain differences among bacterial communities, we used non-metric multidimensional scaling (NMDS) plots of the unweighted Unifrac distances. Unweighted Unifrac distance matrices were also used for PERMANOVA analyses of microbial community data using the adonis function in the vegan package of R [64,66]. A PERMANOVA was used to compare bacterial community structures across all treatment groups based on the OTU composition and examine the relationship between relative abundances of the most abundant phyla or classes, as well as diet, rearing, and sterility variables. Int total, 9,516,032 raw reads were obtained and raw OTU counts were used to calculate the Shannon Diversity and species richness. Then, the species richness and Shannon diversity were compared across treatment sources and treatment conditions [67].

### 2.6. Indicator Species Analysis

We applied an indicator species analysis to detect bacterial families significantly associated (*p* < 0.05) with the two groups, sterile/non-sterile, as well as between garden/lab-reared. We calculated the indicator values using the ‘multiplatt’ function with 9999 permutations in the ‘indicspecies’ R package [68]. Indicator value indices were used for assessing the predictive values of taxa as indicators of conditions present within the different groups [68].

## 3. Results

**Figure 1:**
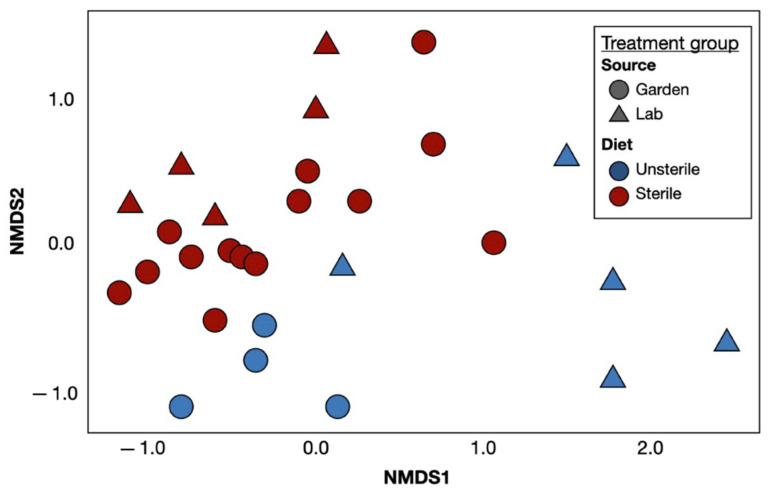
A non-metric multidimensional scaling (NMDS) depiction of unweighted Unifrac distances values illustrates how bacterial communities of *Ambigolimax valentianus* vary by environmental and dietary treatments. Samples of slugs raised under sterile conditions are represented by red, while slugs in unsterile conditions are symbolized by blue; slugs reared in the lab are represented by triangles and slugs from the garden outside of the laboratory are indicated by circles. Garden Unsterile N = 4, Garden Sterile N =14, Lab Unsterile N = 4, Lab Sterile N = 5.

Our PERMANOVA analyses revealed that the structure of the slug bacterial communities was significantly different between dietary treatments, as well as between environments (*p* < 0.001, *p* < 0.001; Figure 1). Both lab-reared and the garden slug’s bacterial communities adapted similarly after combined sterile diet and sterile environmental exposure. Prior to sterile diet and sterile environmental exposure, the lab-reared and garden slug microbiomes mostly did not overlap. These results support our first hypothesis that the bacterial microbiome of *A. valentianus* can be manipulated via changes in their environment and diet.

Despite differences observed in the bacterial microbiome between groups, we detected a likely core microbiome for *A. valentianus* slugs. There were several slug bacterial OTUs conserved across all slug samples (Table 1), which is consistent with our second hypothesis, that a core bacterial microbiome is present and not changed due to experimental perturbations. While some bacterial taxa were shared across all treatments, both sterility (*p* < 0.015) and environment (*p* < 0.007) explained the variation among the total slug bacterial microbiomes.

The indicator species analysis yielded a total of 54 significant taxa (Table S1) across all groups. The sterile slug group produced four significant bacterial species (*p* < 0.01) as well as seven significant bacterial species (*p* < 0.01) for the non-sterile slug group. *Propionibacteriuma acnes* was the most significant (*p* < 0.01) indicator for the garden slug group. Highly significant (*p* < 0.001) indicators for the lab-reared slug group included the bacterial genera *Pigmentiphaga*, *Ochrobactrum*, *Leucobacter*, *Candidatus Solibacter*, and *Luteolibacter* and the bacterial families *Rhodobacteraceae* and *Rhodocyclaceae*.

**Figure 2:**
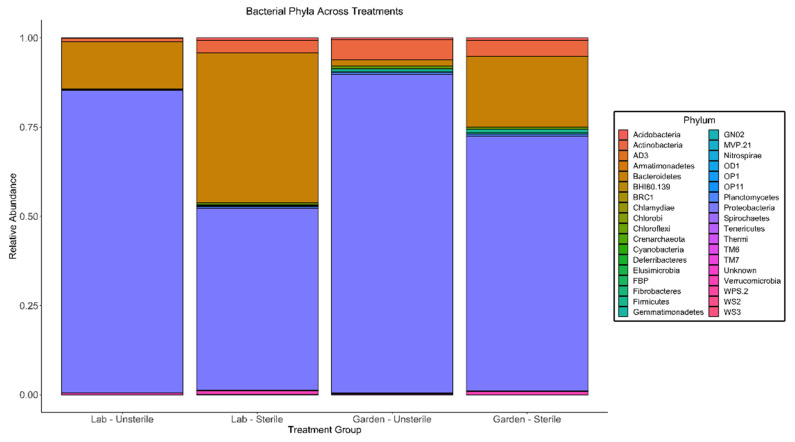
Relative abundances of taxa composing slug bacterial microbiomes among treatment groups, shown at the phylogenetic level of microbial phylum. The relative abundances of microbial phyla in associated treatment groups shifted after exposure to sterile conditions. Garden Unsterile N = 4, Garden Sterile N = 14, Lab Unsterile N = 4, Lab Sterile N = 5.

Proteobacteria have a higher relative abundance in slugs fed the unsterile diet compared to the sterile diet, yet garden slugs appear to contain a higher relative abundance of proteobacteria altogether (Figure 2).

**Figure 3:**
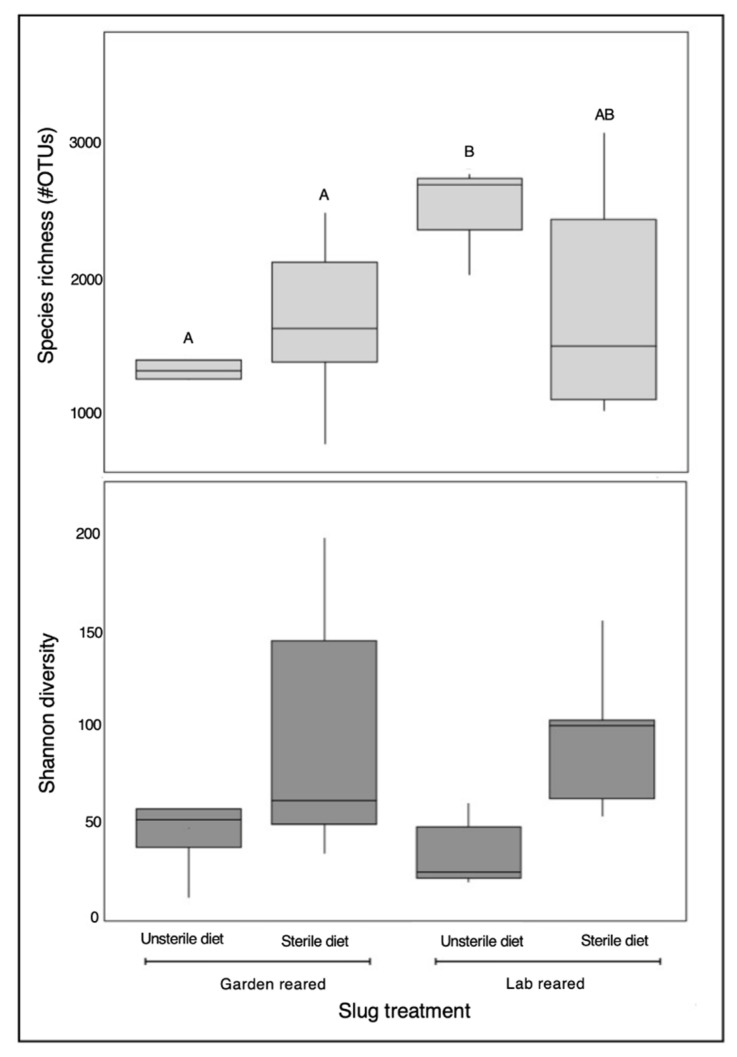
Box plot representation of species richness and Shannon diversity among treatment groups, compared using Wilcoxon tests. Treatments with individual A or B letters are significantly different from one another across treatment groups (*p* < 0.05). Treatments sharing A or B letters are not significantly different from on another across treatment groups. Lab-reared slugs fed an unsterile diet harbored significantly more bacterial taxa in their microbiome than garden-reared slugs. Shannon diversity values were equivalent across all treatment groups. Garden Unsterile N = 4, Garden Sterile N = 14, Lab Unsterile N = 4, Lab Sterile N = 5.

We detected greater species richness in slugs which were reared in the lab that were fed an unsterile diet compared to what was found in slugs reared in the garden, which were fed an unsterile diet (Figure 3). For slugs fed an unsterile diet, we found that bacterial species richness was greater in the lab group than the garden slug group; however, no differences were detected in slug bacterial diversity in these same slug groups. Similarly, slugs reared in the lab that were fed an unsterile diet contained a greater bacterial species richness than slugs reared in the garden and fed a sterile diet (Figure 3). Yet, Shannon diversity values were equivalent across all treatment groups, regardless of where they were reared, or the sterility associated with their dietary inputs (Figure 3).

## 4. Discussion

In this present study, we investigated the impact of diet and environment on the composition and diversity of *A. valentianus’s* bacterial microbiome. We found that bacterial communities of slugs differed between source groups, ostensibly resulting from differences in both diet and environment. Sterility treatments in diet and environment subsequently led to similar shifts in the slug bacterial microbiome regardless of source location. However, we did detect some overlap in bacterial communities across these treatments.

The patterns of overlap in microbial community members across the treatments were more evident in the sterile slug samples. This may indicate that the structure of the bacterial microbiomes of slugs in unsterile habitats may be retained within and from their environment. A large proportion of the environmental bacteria, including those that may be incidental and not specific to the slugs, may have been lost when the slugs were put into similar sterile environments, as evident by the overlap in the core bacterial microbiome.

Previous studies have shown that diet and environment play a role in determining the bacterial microbiome of a variety of invertebrates. A study from Cavalcante et al. [18] showed that a diet of only sugarcane produced a shift on the gut microbial communities of *Achatina fulica*, a land snail. Landry et al. [69] presented evidence that environment, including diet, has a significant effect on the microbial species diversity in the midgut of *Choristoneura fumiferana*, the spruce budworm. Our findings were comparable to the results reported in these and other studies of invertebrate microbiomes. *Ambigolimax valentianus* bacterial microbiomes from groups reared in either the garden or laboratory environments differed initially. Moreover, the microbial communities of these slugs raised in these different environments shifted similarly after exposure to sterile food and environment. Our study provides evidence that slug bacterial microbiomes are malleable and may depend substantially on both diet and environment. Additionally, the shift in the microbial community from these conditions supports our first hypothesis that diet and environment may have an impact on the bacterial microbiome of *A. valentianus*.

Research indicates that the bacterial microbiome of many organisms can be altered by reducing certain key nutrients in their diet [18,21]. Some ingredients in the nutrient agar (peptone, beef/yeast extract) used for the sterile diet possibly provided the slugs with some nutritional value [70,71]; however, it may not have been adequate. It is likely that this dietary restriction may have played a role in the shift of their bacterial microbiome. However, slugs feed on a wide diversity of material varying in nutrient content, which can partially compensate for reduced nutrient intake by adjusting their ingestion and utilization of proteins and carbohydrates [49,50]. This might allow the slug gut bacterial community to be less sensitive to nutritional changes, therefore remaining somewhat balanced despite varying levels of nutritional intake. The variations in the structure of the bacterial microbiome of *A. valentianus* could be attributed to a range of factors. Nonetheless, this research, as well as many other snail and slug studies, has demonstrated that Proteobacteria are the dominant species found among these gastropod microbiomes, regardless of feeding conditions or nutrient uptake (sterile diet, starved, complete diet) [12,13,14,15,16,17,18,19,20,21,22,23,24,25,26]. Further investigation into the nutritional and digestive roles of the bacterial microbiome of *A. valentianus* would provide more insight into the specificity of their associated bacteria.

Some insects that feed on nutrient-deficient diets are associated with endosymbionts that provide them with key nutrients and aid in their overall survival [72,73,74,75]. Slugs do not have a long life expectancy (6 months–2 years) and shorter under laboratory-reared conditions [47,54]. In the field, *A. valentianus* live up to approximately one year [52], but in a separate laboratory experiment, adult *A. valentianus* slugs subsisted on the sterile diet for five months before collection [76], suggesting that (1) the sterile diet provided adequate nutrients or (2) slugs can survive while consuming minimal nutrients. Endosymbionts involved in such relationships with other organisms are within the Proteobacteria phylum [77,78,79,80,81]. Slugs may harbor endosymbionts that aid in the nutrient synthesis critical for their survival. These endosymbionts may also protect the herbivorous slug from chemically defended plants [74,75,82,83]. Further research on the consequences of slug host nutrition and the bacteria involved would be an important step in the development of endosymbiont-based control strategies.

The diet and environment of invertebrates directly or indirectly play substantial roles in shaping their microbiome, such as environmental pressures on resident microbiota and overall survival in the invertebrate gut [1,2]. Slugs are known vehicles of microorganism dispersal, yet the impact of these transient microorganisms on the slug host microbiome is unknown. Future research aimed at determining whether microorganisms acquired by slugs can influence their microbiome or affect their capacity to disperse microorganisms to novel environments—as well as examining if the core bacterial microbiome of slugs develop via vertical transmission—would be especially valuable.

Although there was a core microbiome of shared bacterial taxa isolated from *A. valentianus* slugs across all sequenced slug microbiome samples, all but one of the core bacterial taxa found in *A. valentianus*, *Rhodococcus fascians*, were not identified at the species level. To clarify, unique sequences identified as OTUs were found in all slugs, but those sequences were only identifiable to genera. We discovered eight bacterial families in the core bacterial microbiome of *A. valentianus*, which included bacterial species found in previous studies of gastropod microbiomes. One genus detected across all of our sequenced samples, *Citrobacter*, has not only been found in fecal samples of the slug *Geomalacus maculosus*, but also in the gut of another slug species *Arion ater* [15,17]. *Geomalacus maculosus* also consisted of similar core bacteria, including such genera as *Aeromonas*, *Buttiauxella*, *Citrobacter*, *Kluyvera*, and *Pseudomonas* [15]. We have previously found *Pseudomonas* in the microbiome of *A. valentianus* and *Arion* sp. slugs in our unpublished preliminary work. These findings support our hypotheses that a core microbiome is present in *A. valentianus*.

Similar to our study, Joynson et al. [25] also determined that the majority of the gut microbial community of *Arion ater* corresponded to members of the Enterobacteriaceae and Pseudomonadaceae families [25]. Additionally, the bacterial family *Comamonadacea* was detected within the core bacterial microbiome of *A. valentianus*; previous research illustrated that taxa from *Comamonadacea* can be recovered from the gut of the giant African land snail *Achatina fulica* [24]. Other studies detected genes linked to lignocellulose degradation within the microbiota from the crops of the giant African land snail [16,18]. Some genera in the family *Comamonadaceae* have been directly linked to the degradation of lignocellulose [84], which could have implications for host nutritional status or for amplifying economic crop losses. Determining the extent of bacterial genes associated with lignocellulose degradation in *A. valentianus* could be economically significant and relevant for food security. The diversity of the core bacterial microbiome of *A. valentianus* slugs in this study is slightly more diverse than slug microbiomes analyzed in previous studies [15,17,85]. This could be due to our use of whole slugs in this study, whereas other studies limited their microbiome analyses to specific regions of the slug’s anatomy [15,17,85].

Some of the bacterial taxa found across the *A. valentianus* core bacterial microbiome are putative plant pathogens. For instance, *Erwinia* is a genus containing mostly plant pathogenic species [86,87], and *Rhodococcus fascians* is a plant pathogen which causes leafy gall disease in a variety of plants. Additionally, a subset of other plant pathogens, such as *Pseudomonas viridiflava*, were found in some of our slug samples, but were otherwise absent in others [88].

The indicator species analyses revealed families, genera, and species of bacteria, characteristic of each treatment group individually. In fact, according to our indicator species analyses, the most significant taxon in the sterile groups (slugs fed a sterile diet in a sterile environment) included the families *Aeromonadaceae* and *Cerasicoccaceae* as well as genera *Flavobacterium* and *Mycobacterium*; this could indicate that these taxa may be poor competitors with other members of the non-sterile group. The most highly significant bacterial species found in the non-sterile slug group, *Paracoccus marcusii*, is known to produce astaxanthin, a carotenoid that produces a red/orange pigment which not only provides a variety of plants and animals with their red/orange color but has also been linked to having beneficial (photoprotective, antioxidant, and anti-inflammatory) effects on the skin [89,90,91]. Additionally, *Paracoccus marcusii* was isolated from the white grub, a serious pest of potatoes, in a study that attempted to find entomopathogenic bacteria associated with the grub [92]. In the garden slug group, six bacterial families were identified as indicators. Overall, *Propionibacterium acnes* was the most significant bacterial indicator of the garden group. Although this taxon has been reported as a member of the skin microbiome and is associated with acne pathogenesis [93], previous studies have not identified *Propionibacterium acnes* as a common garden taxon associated with slug’s microbiomes. Many bacterial taxa, across seven bacterial families, were indicative of the lab-reared slug group. Across these seven bacterial families, many taxa, which were previously isolated from a variety of organisms, have been found to be linked to either gut health or gut microbiome. Of these taxa, *Pigmentiphaga* has been isolated from nematodes [94], *Ochrobactrum* from bees, spiders, nematodes, and sand flies [94,95,96,97], and *Rhodocyclaceae* from termites and beetle larvae [98,99]. Often detected within woody plant parts, *Leucobacter* has been found to exhibit mutualistic relationships with keystone soil invertebrates, ostensibly due to its ability to degrade lignocellulose into more labile components and bioavailable nutrient sources [100]. Likewise, *Candidatus Solibacter* has also been associated with decomposing dead wood and peat moss [101,102]. *Luteolibacter*, in the family *Verrucomicrobiaceae*, was identified as part of the core microbiome within fecal samples of *Geomalacus maculosus*, a European protected slug [15]. Thus, based on their ecological roles, interactions with plant or animal hosts, or their physiological adaptations to particular environments or diets, the occurrence of the bacterial indicators may be characteristic of each group; for instance, the presence of particular taxa may correspond with either changes in slug diets (sterile vs non-sterile) or changes in response to their environment (garden and lab-reared).

## 5. Conclusions

Our study provides evidence that bacteria associated with slugs are not only ecologically significant but may also be manipulated by both dietary and environmental changes. Several microorganisms found within our slug bacterial microbiomes have been detected in the guts or feces of other slugs, which could have functional implications for host processes and dietary parameters. Given that invasive slugs can harbor a variety of plant pathogenic microorganisms within their microbiome, their dispersal could have environmental and agricultural implications for both crop health and plant science. The findings from our study suggest that although a small core microbiome remains consistent, the establishment of the slug bacterial microbiome not only varies among individuals but may also be manipulated by dietary and environmental changes. Nevertheless, a better understanding of the slug bacterial microbiome may provide valuable information regarding biotic threats posed by invasive slugs, as well as insight into potential techniques for holistically managing slug populations.

## Figures and Tables

**Table 1:** The Core Bacterial Microbiome—taxa present in all *Ambigolimax valentianus* samples.

Bacterial Genus	Bacterial Family
*Citrobacter*	*Enterobacteriaceae*
*Delftia*	*Comamonadaceae*
*Erwinia*	*Enterobacteriaceae*
*Arthrobacter*	*Micrococcaceae*
*Stenotrophomonas*	*Xanthomonadaceae*
*Pseudomonas*	*Pseudomonadaceae*
*Rhodococcus*	*Nocardiaceae*
*Bacillus*	*Bacillaceae*

## Data Availability

The data presented in this study are openly available through the Dryad repository at https://doi.org/10.6086/D1W39K, accessed on 16 June 2021.

## Supplementary Materials

The following are available online at https://www.mdpi.com/article/10.3390/insects12070575/s1, Table S1: Indicator species, Table S2: Wilcoxon_Diversity.Richness.Res.
